# Amyloid blood biomarker detects Alzheimer's disease

**DOI:** 10.15252/emmm.201708763

**Published:** 2018-04-06

**Authors:** Andreas Nabers, Laura Perna, Julia Lange, Ute Mons, Jonas Schartner, Jörn Güldenhaupt, Kai‐Uwe Saum, Shorena Janelidze, Bernd Holleczek, Dan Rujescu, Oskar Hansson, Klaus Gerwert, Hermann Brenner

**Affiliations:** ^1^ Department of Biophysics Ruhr‐University Bochum Bochum Germany; ^2^ Division of Clinical Epidemiology and Aging Research German Cancer Research Center (DKFZ) Heidelberg Germany; ^3^ Department of Clinical Sciences Lund University Lund Sweden; ^4^ Saarland Cancer Registry Saarbrücken Germany; ^5^ Department of Psychiatry, Psychotherapy and Psychosomatics University of Halle Halle Germany; ^6^ Memory Clinic Skåne University Hospital Malmö Sweden; ^7^ Network Aging Research (NAR) University of Heidelberg Heidelberg Germany

**Keywords:** Alzheimer's disease diagnosis, amyloid‐β in blood plasma, BioFINDER, ESTHER, immuno‐infrared‐sensor, Biomarkers & Diagnostic Imaging, Neuroscience

## Abstract

Alzheimer's disease (AD) is currently incurable, but there is general agreement that a minimally invasive blood biomarker for screening in preclinical stages would be crucial for future therapy. Diagnostic tools for detection of AD are either invasive like cerebrospinal fluid (CSF) biomarkers or expensive such as positron emission tomography (PET) scanning. Here, we determine the secondary structure change of amyloid‐β (Aβ) in human blood. This change used as blood amyloid biomarker indicates prodromal AD and correlates with CSF AD biomarkers and amyloid PET imaging in the cross‐sectional BioFINDER cohort. In a further population‐based longitudinal cohort (ESTHER), the blood biomarker detected AD several years before clinical diagnosis in baseline samples with a positive likelihood ratio of 7.9; that is, those who were diagnosed with AD over the years were 7.9 times more likely to test positive. This assay may open avenues for blood screening of early AD stages as a funnel for further more invasive and expensive tests.

## Introduction

Alzheimer's disease (AD) is a brain disorder whose hallmarks are amyloid plaques and neurofibrillary tangles (Blennow *et al*, 2006; Zhao *et al*, 2012; Wang & Mandelkow, 2016). It is believed that up to 15–20 years prior to clinical symptoms of AD (Bateman *et al*, 2012; Rowe *et al*, 2013), the secondary structure of amyloid‐β (Aβ) peptides alters its folds from “healthy” disordered or α‐helical to “pathological” β‐sheet‐enriched secondary structures (Sarroukh *et al*, 2011). Such β‐sheet‐enriched structures aggregate and can form soluble toxic oligomers, seeds, and finally macroscopically visible amyloid plaques, which are thought to contribute to AD neurodegeneration (Cavallucci *et al*, 2012; Zhao *et al*, 2012; Jucker & Walker, 2013). Hence, research diagnostic criteria in AD recommend the use of biomarkers of amyloidosis (McKhann *et al*, 2011; Sperling *et al*, 2011; Jack *et al*, 2016). Established amyloid biomarkers are Aβ‐binding ligands used in positron emission tomography (PET), a costly and time‐consuming technique, and assays to measure Aβ_42_, total‐tau (ttau) and phosphor‐tau (ptau) in cerebrospinal fluid, which require an invasive procedure (Blennow *et al*, 2015).

**Figure 1:**
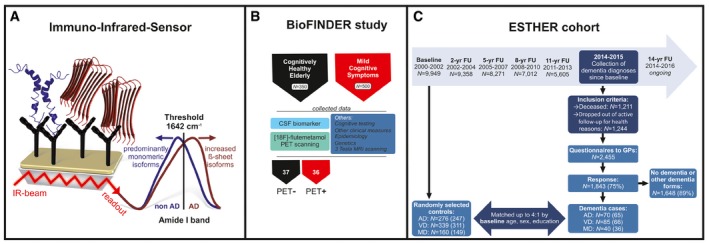
Schematic overview of the immuno‐infrared‐sensor, the assay read‐out, and BioFINDER and ESTHER study design The immuno‐infrared‐sensor simultaneously monitors the secondary structure distribution of all soluble Aβ peptides in blood plasma caught by monoclonal antibodies (mAb) covalently attached to the sensor surface (schematic simplified representation; mAb A8978 is raised against the middle epitope (aa13–28) of Aβ). If the marker band (amide I) is dominated by disordered or α‐helical monomeric isoforms, the patients would be diagnosed as non‐AD (blue). If β‐sheet isoforms are enriched (red), the amide I signal is shifted below the threshold (1,642 cm^−1^), indicating AD.Plasma samples from the cross‐sectional study (BioFINDER) were measured with the immuno‐infrared‐sensor and used to differentiate between 37 healthy elderly people (all PET negative) and 36 prodromal AD (MCI) patients (all PET positive). The clinical record of participants within this cohort comprised cognitive testing, CSF biomarker assessment, other clinical measures, 3 Tesla MRI, genetics, epidemiology and [^18^F]‐flutemetamol PET scanning.Nested case–control study based on the ESTHER cohort study as of January 2016. Baseline blood plasma samples from the year 2000–2002 were measured in the same way as samples from the BioFINDER cohort. AD = Alzheimer's disease; VD = vascular dementia; MD = mixed dementia are investigated. Other dementia forms include frontotemporal dementia and other dementias. GPs = general practitioners; yr = year; FU = follow‐up. The number of randomly selected controls and dementia cases reflects the original study design; the measured sample sizes are depicted in brackets.

Recently, we developed an immuno‐infrared‐sensor (WO 2015121339 A1) that monitored the secondary structure change of Aβ peptides. The sensor is an antibody‐based (immuno) method to extract all Aβ peptides from CSF and blood samples and spectroscopically senses the secondary structure distribution of extracted soluble Aβ peptides in the infrared (Fig 1A; Nabers *et al*, 2016a,b). As compared to established ELISA tests for Aβ detection, the immuno‐infrared‐assay does not measure the absolute biomarker concentration but the frequency shift in the infrared as a relative measure. Thus, the relative measurements are in principal more robust against concentration fluctuations caused by biological variances.

We showed that the change in the Aβ peptides secondary structure is a reliable blood biomarker for severe AD stages (Nabers *et al*, 2016a). As expected, AD patients at these stages showed an overall shift to β‐sheet‐enriched secondary structures. The immuno‐infrared‐sensor specifically extracts all soluble Aβ forms from blood plasma under physiological conditions independent of the respective secondary structure. The read‐out is the frequency of the secondary structure‐sensitive amide I absorbance band, which reflects the average secondary structure distribution of all soluble Aβ forms (see Fig 1A). In β‐sheet conformations, comprising diverse oligomeric and (pre‐) fibrillary Aβ species, the amide I band is downshifted relative to α‐helical or disordered monomeric isoforms (Fig 1A). The relative small amide I band of Aβ is elucidated from the large background absorbance by difference spectroscopy (Garczarek & Gerwert, 2006; Nabers *et al*, 2016a,b). An experimentally determined threshold frequency separates the amide I marker band in β‐sheet‐enriched species from unfolded and α‐helical isoforms (schematically shown in Fig 1A). In summary, if the maximum position of the marker band shifts below the threshold, it indicates AD, because the average secondary structure distribution is shifted towards β‐sheet‐enriched species.

In a previous cross‐sectional clinical study (Nabers *et al*, 2016a) that included neurochemical markers from cerebrospinal fluid (CSF) (such as Aβ(40), Aβ(42), Aβ(42/40), phospho‐tau, total‐tau), the sensor was applied to 141 patients with moderate‐to‐severe AD stages. In the previous study, the immuno‐infrared‐assay showed a sensitivity of 94% and a specificity of 88% for CSF analyses. However, even more interesting it showed a sensitivity of 75%, and a specificity of 88% for blood plasma analyses (Nabers *et al*, 2016a). This demonstrated that the Aβ secondary structure change could be used as a blood biomarker for severe AD stages.

## Results

### The amyloid‐β secondary structure distribution in blood plasma of prodromal AD (BioFINDER)

Since the structural change of Aβ is proposed to take place 15–20 years before clinical onset, here, we investigated whether this marker band downshift below the threshold could also serve as a blood biomarker to identify early AD stages.

**Figure 2:**
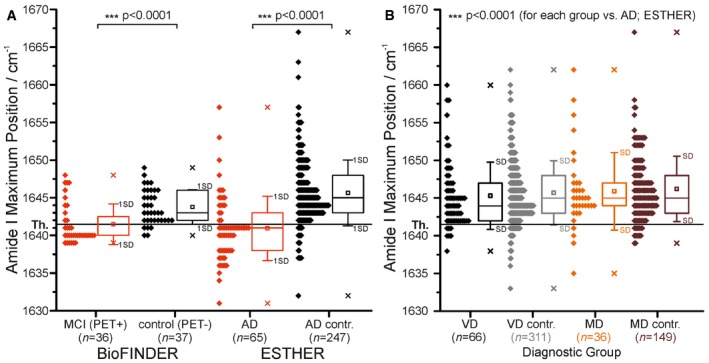
The Aβ amide I band absorbance maxima as recorded from baseline blood plasma samples from the BioFINDER and the ESTHER study are shown as diamonds The threshold at 1,642 cm^−1^ (solid horizontal line) separates AD cases (MCI (PET positive), BioFINDER; AD, ESTHER) from controls (control (PET negative), BioFINDER; AD contr., ESTHER).The threshold at 1,642 cm^−1^ also separates AD cases (for AD, see A) from VD, MD cases and respective controls. Only the AD group demonstrated significantly lower maxima below the threshold. Therefore, the biomarker band may provide a differential diagnosis. All diagnostic groups differed significantly (*P *<* *0.0001) from the AD group (ESTHER).Data information: In box plots, 25/50/75% quantiles are shown as horizontal lines, the observed minimum/maximum values as (×), the average amide I band position as square, and ± standard deviation as whiskers. Significant group differences are indicated by *P*‐values (two‐sided nonparametric Kruskal–Wallis analysis of variance test) and by asterisks: *P <* *0.05, **P <* *0.01, ***P <* *0.001.

In order to answer these questions, we first studied whether the Aβ secondary structure distribution was altered in plasma during the prodromal stages of AD. From the prospective Swedish BioFINDER study, we included 36 participants with prodromal AD, that is, non‐demented patients with mild cognitive impairment (MCI) that had abnormal amyloid [^18^F]‐flutemetamol PET scans, as well as 37 cognitively healthy controls that had normal amyloid PET scans (Fig 1B). In the original study design, 40 prodromal AD cases versus 40 cognitively healthy were planned, but four and three samples, respectively, showed an insufficient performance, and thus, sample analysis was not possible. All plasma samples collected from the subjects of the BioFINDER study were analysed using the immuno‐infrared‐sensor (samples were blinded). The amide I biomarker band was significantly lower (*P* < 0.001) in cases with prodromal AD compared with the control group (Fig 2A).

**Figure 3:**
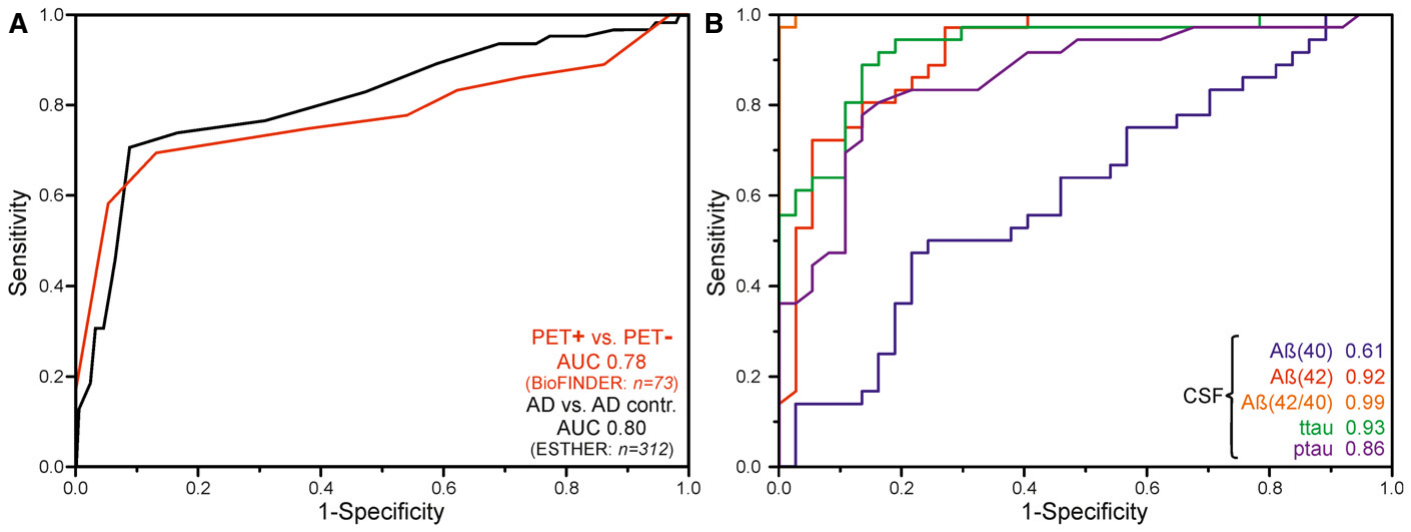
ROC curve analyses of the BioFINDER and the ESTHER data set ROC curves for prodromal PET‐positive cases (*n *=* *36) vs. healthy elderly PET‐negative subjects (*n *=* *37) (BioFINDER) and AD (*n *=* *65) vs. AD control (*n *=* *247) differentiation (red) (ESTHER, Germany) were obtained by variation of the threshold frequency between 1,630.5 and 1,660.5 cm^−1^. An AUC of 0.78 (0.68–0.88, 95% CI) for the BioFINDER cohort and AUC of 0.80 (0.76–0.84, 95% CI) were achieved for the ESTHER cohort. Using a cut‐off (threshold) of 1,642 cm^−1^ as the lower limit for test negativity yielded a sensitivity of 71% at 91% specificity for the ESTHER study (black) and a sensitivity of 69% at specificity of 86% for the BioFINDER study (red).ROC curves for prodromal PET‐positive cases and healthy elderly PET‐negative subjects differentiation (BioFINDER, Sweden) were calculated for CSF biomarkers such as Aβ(40) (blue), Aβ(42) (red), Aβ(42/40) ratio (orange), ttau (green) and ptau (pink). AUC values are presented. Source data are available online for this figure.

However, the average amide I downshift is not as large as in the severe state, indicating less enrichment of β‐sheet as expected for this very early state. Using the diagnostic threshold of 1,642 cm^−1^, the controls and prodromal AD subjects in the BioFINDER study were differentiated with a sensitivity of 69% and specificity of 86%. An AUC of 0.78 (0.68–0.88, 95% CI) was calculated for the differentiation between prodromal AD cases and controls (Fig 3A). This shows that the blood biomarker is also able to differentiate between early stages of Alzheimer's disease and cognitively healthy individuals. However, the performance is not as good as for severe stages with sensitivity of 75%, and a specificity of 88%. The performance might be improved significantly in the future by using an optimised antibody for the assay as preliminary experiments have been shown.

Further, the amide I frequency of Aβ in plasma samples showed significant correlations with the levels of CSF AD markers in the BioFINDER study: Aβ(42) (*P*‐value = 4 × 10^−4^, *r*_s_ = 0.401), Aβ(42/40) ratio (*P *=* *4 × 10^−4^, *r*_s_ = 0.407), total‐tau (*P *=* *8 × 10^−4^, *r*_s_ = −0.382), phospho‐tau (*P *=* *0.146, *r*_s_ = −0.172).

**Figure EV1:**
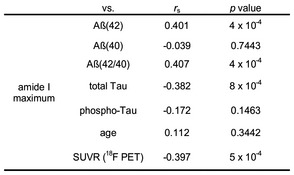
Correlation between Aβ from blood plasma and CSF or PET (BioFINDER) Summary of the Spearman rank correlation coefficients (*r*_s_) and their *P*‐values between the amide I maximum positions of Aβ from EDTA plasma, neurochemical biomarkers from CSF and SUVR values from ^18^F PET imaging.

**Figure 4:**
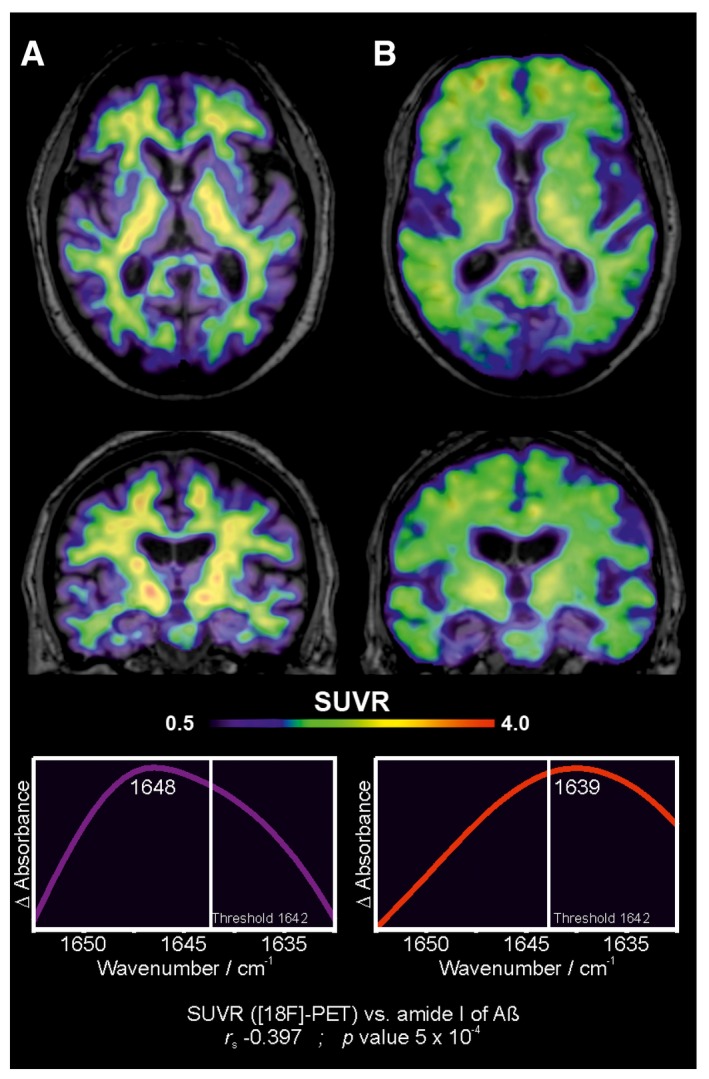
Correlation between Aβ deposition in the brain (PET) and the Aβ secondary structure distribution in plasma The figure depicts transaxial and coronal sections of [^18^F]‐flutemetamol PET scans obtained from a cognitively healthy control (BioFINDER). The PET scan shows non‐specific binding of the radioligand in the white matter. The corresponding infrared amide I band for Aβ from this patient's EDTA‐blood plasma is presented below. The PET and infrared data correlate significantly with each other (*r*_s_ = −0.397, *P*‐value = 5 × 10^−4^) (see Fig EV1).The figure depicts transaxial and coronal sections of [^18^F]‐flutemetamol PET scans obtained from a patient with prodromal AD (BioFINDER). The PET scan shows clearly increased cortical binding of [^18^F]‐flutemetamol. The corresponding infrared amide I band for Aβ from this patient's EDTA‐blood plasma is presented below. The PET and infrared data correlate significantly with each other (*r*_s_ = −0.397, *P*‐value = 5 × 10^−4^) (see Fig EV1).

The [^18^F]‐flutemetamol standardised uptake value ratio (SUVR) of a cortical composite region correlated significantly with the IR‐marker band in the plasma (*P *=* *5 × 10^−4^, *r*_s_ = −0.397397; Fig EV1). Figure 4 illustrates the correlation between the [^18^F]‐flutemetamol PET image and the infrared amide I band for a control and a prodromal AD subject.

All neurochemical and demographic data of the 73 BioFINDER participants are summarised in Appendix Table S2.

Thus, we were able to correlate our plasma biomarker with neurochemical biomarkers and amyloid PET scans. The sensitivity and specificity obtained in blood were lower than those obtained in CSF (Fig 3B), and this currently prevents the employment of the blood test as a stand‐alone diagnostic instrument, but it might allow its use in a two‐stage screening process including a combination of the amyloid blood biomarker with neurochemical CSF biomarkers of AD and/or PET scanning. Moreover, this test might be used in combination with other blood tests for AD that may become available in the future and that, ideally, should also focus complementary on tau filaments, a second hallmark in AD (Wang & Mandelkow, 2016). Particularly, the blood test could be used to preselect individuals in early disease stages for additional lumbar puncture and PET scanning.

### The amyloid‐β secondary structure distribution in blood plasma of early state/preclinical AD (ESTHER)

In the next step, we investigated whether this marker band downshift below the threshold could also serve as a blood biomarker to identify AD years before clinical diagnoses in the general population. Therefore, we measured plasma samples from a population‐based longitudinal cohort of older adults from Germany (ESTHER study) started in the years 2000–2002. In a nested case–control study approach embedded in the longitudinal ESTHER study (Saum *et al*, 2015) and comprising 970 participants (see online methods for more details), we identified 195 participants diagnosed with dementia during follow‐up, and randomly selected approximately four times as many controls without a dementia diagnosis. These controls were frequency matched to AD (*n* = 70), vascular dementia (VD; *n* = 85) and mixed dementia (MD; *n* = 40) cases by baseline age, sex and educational level (Fig 1b). For this study, we excluded 70 participants with haemolytic plasma samples, 19 cases for whom the suspected dementia diagnosis could not be confirmed by further medical records and seven purported controls for whom dementia diagnosis was later reported. This left 167 cases (65 AD, 66 VD, 36 MD) and 707 controls (247 AD, 311 VD, and 149 MD) for Aβ‐analyses (Appendix Table S1).

The ESTHER cohort is a representative cohort not specifically designed for AD research; hence, diagnoses of AD in this group are not necessarily supported by invasive CSF biomarkers nor PET scanning (McKhann *et al*, 2011). However, blood plasma samples used for the present analyses were collected at baseline (2000–2002), several years before clinical diagnosis of AD. Diagnoses of AD were made or confirmed at later stages of the disease in the follow‐up process of this longitudinal study. At these later stages, clinical diagnoses are more accurate even without neurochemical marker assessment and PET scanning (Beach *et al*, 2012; Martinelli *et al*, 2014). Specifically, mean follow‐up time between blood collection and AD diagnosis was 8 years as listed in detail in the Appendix Table S3. Also, the carriage of the *APOE* e4 risk allele for AD in the ESTHER cohort was much more common in AD cases than in AD controls (45.8% vs. 21.7%. $P{\text{‐value}}_{\chi^{2}\;\mathrm{test}}$ = 0.0002) (Appendix Table S1) and the risk allele prevalence in AD cases is in agreement with the estimates reported in other German studies on AD [pooled prevalence estimates for the e4 allele: 53.0% (44.9–61.2) (Ward *et al*, 2012)]. Mean ages at baseline of AD cases vs. the respective controls were 68.7 vs. 68.5 years. The majority of AD cases and AD controls were women (61.5% and 62.4%, respectively) and had low levels (9 years or less) of school education (86.2% and 83.8%, respectively).

The immuno‐infrared‐sensor extracted the total Aβ fraction from baseline plasma samples from each subject and assayed them in a blind manner. Values of the amide I biomarker band were significantly lower (*P* < 0.001) for AD cases as compared with the control group (Fig 2A). This shows that the amide I biomarker was able to detect AD in individuals without dementia symptoms on average 8 years before clinical diagnosis of AD.

The downward shift below the threshold of the amide I band to 1,641 cm^−1^ (median 1,641 cm^−1^) was seen almost exclusively for AD cases but not for the other dementia forms and controls (Fig 2B).

This might suggest that the β‐folding of Aβ does not appear in the early stage of other dementias like MD or VD. Nevertheless, we could not exclude some misclassification of MD which might be VD. These preliminary results, indicating that the blood test might also discriminate between AD and VD, have to be validated in another longitudinal AD cohort in whom the diagnosis is additionally confirmed by neurochemical markers and PET scanning. To our knowledge, a blood test, which differentiates between AD, VD and MD is also not available to date.

A receiver operator characteristics (ROC) curve with an AUC of 0.80 (0.76–0.84, 95% CI) characterised the quality of the AD discrimination (Fig 3A). A threshold of 1,642 cm^−1^ yielded a sensitivity of 71% at 91% specificity for AD/AD control differentiation (22 false positives out of 247 AD controls). The overall diagnostic accuracy is 86%. The diagnostic performance indicators in ESTHER (sensitivity, specificity, AUC) were slightly higher and in agreement within the 95% confidence interval with the BioFINDER study (Fig 3A). However, in order to estimate the diagnostic evidence of the prescreening test the likelihood ratios are more appropriate measures. The positive likelihood ratio (LR^+^) was determined with 7.9 (ESTHER) indicating that a positive test result occurs about eight times more frequently for people who develop AD over years compared to those who do not in the general population. Thus, the test showed a good diagnostic evidence (Jaeschke *et al*, 1994) to identify AD before clinical diagnosis in the general population.

## Discussion

The ESTHER results seem to confirm that the test may be used as preselection funnel in a two‐step diagnostics as suggested above for the BioFINDER study. In a prescreening set‐up, individuals with high risk for AD can be identified by the immuno‐infrared‐assay, and in a second step, neurochemical marker assessment and/or PET scanning may be used in order to increase the specificity. For a two‐step diagnostics, the sensitivity could be increased even to 77% at the expense of 69% specificity by up‐shifting the threshold from 1,642 cm^−1^ to 1,644 cm^−1^ (ESTHER). Thus, about 80% of individuals could be preselected for further testing, and about 30% of true negative subjects would wrongly undergo lumbar puncture or PET scanning. However, a significant number of false‐negative subjects will also develop clinical symptoms over years. As long as there is no treatment for preclinical AD, our test result has no implications for these false‐negative AD subjects. Focusing on the Aβ structural change as a marker for the early diagnosis of AD has the great advantage of targeting the molecular pathomechanism of the normal to AD transition, which is known to initiate up to 20 years before clinical manifestation of AD (Bateman *et al*, 2012; Rowe *et al*, 2013). These characteristics allow us to overcome the shortcomings of other non‐brain‐specific blood markers. These include the lack of a clear understanding of their relationship to earliest AD stages and the possibility of being influenced by comorbidities which are common among people with dementia (Fiandaca *et al*, 2014, 2015; Henriksen *et al*, 2014; Mapstone *et al*, 2014; Zhao *et al*, 2015; O'Bryant *et al*, 2017). Also, the finding that Aβ β‐folding appears in the blood could lead to deeper insights into Aβ pathophysiology, including damaged blood–brain barrier and generation of Aβ from cells outside the brain and, possibly, even in comorbidity with other diseases like diabetes. However, many of the factors that may play a role in Aβ pathophysiology in blood are out of scope of this manuscript and further studies with the immuno‐infrared‐sensor should specifically address this issue in more detail.

A limitation of this blood test is that Aβ β‐misfolding may be present in some persons without dementia and be absent in some AD patients; hence, false positives and false negatives are intrinsic to amyloid biomarkers (Yang *et al*, 2012; Fiandaca *et al*, 2014; Henriksen *et al*, 2014; Jansen *et al*, 2015). However, correlations between Aβ plasma tests—either Aβ misfolding as shown here or Aβ_42_ decrease (Ovod *et al*, 2017; Nakamura *et al*, 2018)—and PET scanning demonstrate that there is a direct link between Aβ alterations in plasma and Aβ burden in the brain and that this is not a peripheral effect. This is a very encouraging result for all future plasma tests.

Nevertheless, the use of the immuno‐infrared‐sensor as an initial screening funnel to identify people who should undergo further diagnostics and eventually take part in clinical trials on therapeutics targeting Aβ misfolding (Sevigny *et al*, 2016) might already be an important step forward because subjects with early AD stages are hard to identify. To our knowledge, there is today no other plasma test available, which has been tested both in an AD research cohort and in the general population.

In order to receive clinical utility in the future, the performance of the immuno‐infrared‐test has to be improved and the assay needs to be validated in a multicentre clinical study especially designed for AD like NIH‐ADNI. Therefore, we need to develop an optimised antibody and standard operating procedures that guarantee a constant and increased performance of the assay in a multicentric set‐up. Even though the immuno‐infrared‐assay was applied to blood plasma from different cohorts with different sample handling protocols demonstrating a robust performance, the assay needs to be further validated in different clinical set‐ups to investigate potential effects of sample handling. For future applications, a standard operation procedure will also be followed for sample collection. Therewith, the amyloid marker might be a valuable instrument as a first minimal‐invasive and less costly step to select participants who should undergo more invasive CSF analyses and costly PET diagnostics. Compared to other blood tests (Ovod *et al*, 2017; Nakamura *et al*, 2018), unique features of this assay are (i) the label‐free detection of the misfolding of Aβ in blood plasma, (ii) the simple procedure, (iii) the low sample volume and (iv) the low cost. Moreover, we determined the secondary structure change of Aβ in plasma which is a direct measure as compared to other methods such as ELISA or mass spectrometry which measured the absence of Aβ_42_ or the ratio between Aβ_42/40_. These methods are limited to measure the consequences of Aβ burden in the brain in CSF or blood while the immuno‐infrared‐assay detects the initial misfolding which occurs 15–20 years before clinical manifestation. Thus, the immuno‐infrared‐assay has the potential to detect AD earlier than Aβ burden tests.

Furthermore, our results could also be of great value for basic research since the identified individuals contribute to get a deeper understanding of the pathophysiological mechanisms triggering AD and might reveal important information by following up individuals with Aβ misfolding over the years.

Promising effects have recently been obtained with an immunotherapy treatment of early AD with a monoclonal antibody against the Aβ peptide (Sevigny *et al*, 2016). The present result supports the finding that the Aβ peptide is crucial in the early onset of AD and its β‐folding provides a possible prescreening instrument for this onset. While further improvement of this blood assay is needed, including further generalisation and validation by larger and well‐established Alzheimer's longitudinal cohorts focusing on preclinical AD, our immuno‐infrared‐sensor paves the way towards a minimal‐invasive blood screening assay for early AD and it shows a novel innovative approach to detect early states of misfolding diseases, which could be extended in the future to other diseases like Parkinson's disease using antibodies against α‐synuclein.

## Materials and Methods

The experiments conformed to the principles set out in the WMA Declaration of Helsinki and the Department of Health and Human Services Belmont Report.

### The ESTHER epidemiological cohort study

The ESTHER cohort study is a population‐based longitudinal study of older Caucasian adults conducted in the German state of Saarland in order to assess the chances for early detection and possible prevention of various chronic diseases, including dementia. Between July 2000 and December 2002, during routine health screening examinations (which are offered free of charge every 2 years to adults above age 35 in Germany), the majority of general practitioners (56%) operating in Saarland recruited 9,949 women and men aged 50–75 years and provided medical information. Participants completed standardised self‐administered health questionnaires and provided blood samples, including heparin plasma samples, which were stored at −80°. Follow‐up questionnaires, medical records, and biological samples were collected after 2, 5, 8, 11, and 14 years. The ESTHER study was approved by the Ethics Committee of the Medical Faculty of the University of Heidelberg and of the Physicians’ Board of Saarland, and all participants gave written informed consent. According to the study protocol, results of the laboratory analyses, which were conducted in a fully blinded manner, were not communicated to the study participants.

In 2014–2015, we asked general practitioners to provide us with detailed information on potential dementia diagnoses (overall and according to type of dementia), including the medical records of medical specialists (neurologists, psychiatrists, memory clinics) for ESTHER participants who had died (*n* = 1,211) or terminated active participation in the study due to health reasons (*n* = 1,244) in order to identify cases that had developed clinically manifest dementia since baseline recruitment. We first focused on ESTHER participants who had dropped out of the cohort upon the assumption that manifest dementia would prevent active participation in cohort studies. Hence, focusing on participants who were no longer active allowed for an efficient collection of dementia cases and limited attribution bias.

As of January 2016, medical information on dementia diagnoses could be obtained for the majority of participants (*n* = 1,843), of whom 212 (11.5%) and 41 (2.2%) were reported to have a certain or suspected diagnosis of dementia, respectively. Diagnoses of Alzheimer's disease (AD), vascular dementia (VD), and mixed dementia (MD) were explicitly reported for 70, 85, and 40 participants, respectively; three cases had a diagnosis of frontotemporal dementia, and for 55 participants, the type of dementia was unspecified. The exact date of diagnosis of dementia was recorded in total for 176 (90.3%) AD, VD, and MD cases and, specifically, for 153 (91.6%) AD, VD and MD cases with Aβ analysis.

The documents and medical records indicated a large heterogeneity in the extent of diagnostic procedures taken for both the initial and differential diagnoses of dementia. Out of 65 AD cases, 45 (69%) were explicitly referred from general practitioners to a medical specialist including neurologists, psychiatrists, memory clinics for dementia diagnosis. While some clinical specialists only made a diagnosis of AD if neuropsychological testing, clinical symptoms, and results from brain imaging techniques were all available, other specialists relied exclusively on core clinical criteria such as insidious onset, cognitive decline, amnestic presentation with no evidence that other pathologies could explain these symptoms (McKhann *et al*, 2011). In at least one case, the medical specialist documented that the patient himself refused to undergo brain imaging. This implies that not all ESTHER patients whose blood samples were part of our cohort analysis were diagnosed with the same diagnostic standard, which reflects routine clinical practice.

Within the ESTHER cohort, we designed a nested case–control (NCC) study and included all reported AD, VD, and MD cases as of January 2016, where the response rate of the GPs reached a plateau. We randomly selected the controls from strata defined by age, sex, educational level as matching factors (frequency matching), so that cases and controls were similar with regard to these relevant confounding factors. In order to increase statistical power, we chose more than one control per case, but we did not exceed the 4:1 ratio, since it has been demonstrated that a further increase of the ratio only leads to trivial improvements in precision and power (Wacholder *et al*, 1992). Controls were selected randomly from the whole ESTHER cohort using the “surveyselect” procedure included in the statistical software SAS^®^ version 9.4 (SAS^®^ Institute Inc., Cary, NC, USA).

Measurement of the *APOE* genetic polymorphism and availability of additional medical data among participants of the ESTHER study also allowed an indirect verification of the correctness of dementia diagnoses. In fact, the distribution of *APOE* genetic polymorphism both among AD cases and among controls is in agreement with other German studies (Hong *et al*, 2009; Ward *et al*, 2012). The high prevalence of *APOE* e4 carriers among AD cases (45.8%) as compared to AD controls (21.7%), as well as to VD diagnoses and MD diagnoses (31.6% and 20.6%, respectively), supports the correctness of AD diagnoses and their differentiation from other forms of dementia. Likewise, the high baseline prevalence of history of cardiovascular diseases among VD cases (25.0%) as compared to VD controls (12.2%) and AD cases (8.1%) supports the correctness of VD diagnoses and its differentiation from AD cases (Appendix Table S1).

Frozen baseline (2000–2002) blood from NCC participants tracked with an identification number (ID) was sent to the Department of Biophysics at the Ruhr‐University of Bochum (hereafter, the “Bochum group”) from the Department of Clinical Epidemiology and Aging Research (DKFZ) located in Heidelberg (hereafter, the “Heidelberg group”). The link between identification number and dementia status as collected over the follow‐up period of the ESTHER study was only known to the members of the Heidelberg group, who were not involved in the Aβ analysis, but not to the Bochum group, who performed the analysis. The assay was therefore performed in a blinded manner solely by the Bochum group. At the termination of the Aβ analysis, the Bochum group sent to the Heidelberg group a file that included the value of the amide I biomarker band for each ID number. Only at this point did the Heidelberg group disclosed to the Bochum group the link between ID numbers and dementia status.

### The BioFINDER study

The BioFINDER study, similarly to the ESTHER study, strictly followed study protocols that were carefully examined and fully approved by the responsible ethics boards and, specifically, by the Regional Ethics Committee in Lund, Sweden. Written informed consent was obtained from all study participants and/or their relatives. The study population stemmed from the prospective and longitudinal Swedish BioFINDER study where participants were recruited at three memory outpatient clinics in Sweden between 2010 and 2014 (further information is available at: http://www.biofinder.se). Briefly, clinical assignment of MCI and cognitively healthy people was performed after patient recruitment at baseline based on cognitive testing, other clinical measures such as Hospital Anxiety and Depression Scale (HADS), functional activities questionnaire (FAQ), or Unified Parkinson Disease Rating Scale (UPDRS), CSF biomarkers (e.g. Aβ(42), total‐tau, phospho‐tau), MRI/CT, PET, genetics (e.g. *APOE* genotype) and epidemiological factors (e.g. family history, education, diabetes, depression). Forty cognitively healthy elderly, who were amyloid ([^18^F]‐flutemetamol) PET negative, and 40 non‐demented patients with mild cognitive impairment, who were amyloid PET positive, were included in the present study. The research subjects were thoroughly assessed for their cognitive complaints by physicians with special interest in dementia disorders. The inclusion criteria for mild cognitive impairment were as follows: (i) objective cognitive impairment; (ii) not fulfilling the criteria for dementia; (iii) a Mini‐Mental State Examination (MMSE) score of 24–30 points; (iv) age 60–80 years; and (v) fluent in Swedish. The exclusion criteria were as follows: (i) cognitive impairment that without doubt could be explained by a condition other than prodromal dementias; (ii) severe somatic disease; and (iii) refusing lumbar puncture or neuropsychological investigation. Cognitively normal controls were recruited from the population‐based Malmö Diet Cancer study. Subjects were eligible for inclusion if they (i) were aged ≥ 60 years old, (ii) scored 28–30 points on the Mini‐Mental State Examination (MMSE) at the screening visit, (iii) did not have cognitive symptoms as evaluated by a physician, (iv) were fluent in Swedish, (v) did not fulfil the criteria of MCI or any dementia. The exclusion criteria were as follows: (i) presence of significant neurologic or psychiatric disease (e.g. stroke, Parkinson's disease, multiple sclerosis, major depression), (ii) significant systemic illness making it difficult to participate, (iii) refusing lumbar puncture and (iv) significant alcohol abuse. In the present study, we only included cognitively healthy cases with normal [^18^F]‐flutemetamol PET scans, as well as MCI cases with abnormal PET scans (see below). For both diagnostic groups, 40 subjects that fulfilled these criteria were randomly selected from the total sample collective and sent to Bochum. Therefrom, 37 healthy controls and 36 prodromal AD cases could be successfully analysed with the immuno‐infrared‐sensor.

Blood and CSF samples were collected on the same day and at the same time of day (plasma was obtained within 15 min of CSF sampling) in the year 2010, when the BioFINDER cohort study was initiated. For plasma collection, blood was drawn into tubes containing EDTA as an anticoagulant. After centrifugation (2,000 *g*, 4°C, 10 min), 1 ml of plasma samples was aliquoted into polypropylene tubes and stored at −80°C. The procedure and analysis of the CSF followed the Alzheimer's Association Flow Chart for CSF biomarkers (Blennow *et al*, 2010). Lumbar CSF samples were collected at the three centres and analysed according to a standardised protocol (Palmqvist *et al*, 2014). CSF samples were centrifuged (2,000 *g*, 4°C, 10 min) after collection, and 1 ml was aliquoted into polypropylene tubes followed by storage at −80°C. Both plasma and CSF underwent one freeze–thaw cycle when 200 μl of samples was further aliquoted into protein LoBind tubes and then stored at −80°C pending biochemical analyses.

### Laboratory procedures

#### Secondary structure distribution of Aβ in plasma samples (ESTHER and BioFINDER)

The total Aβ fraction was extracted out of 50 μl plasma samples from AD cases and controls. For ATR‐FTIR analyses (attenuated total reflection Fourier transform infrared), a Vertex 70V FTIR‐spectrometer (Bruker Optics GmbH, Ettlingen, Germany), equipped with a liquid N_2_ cooled mercury cadmium telluride (MCT) detector, and an external MIR (Middle Infrared) source was used. The ATR unit (Specac Ltd., Slough, England) was inserted into the sample compartment of the spectrometer and adjusted to a 45° incidence angle. The internal sample compartment was purged with dry air (Purge air generator Model 75‐62‐12VDC, Parker Hannifin Corp., Haverhill, MA, USA). The set‐up was completely automated and parallelised by in‐house developments for a higher throughput as reported by Nabers *et al* (2016a) and to maximise the reproducibility of the sensor performance.

Sensor surface modification was performed as described in detail previously (Nabers *et al*, 2016a,b). Briefly, the polished Germanium internal reflection element [Ge‐IRE, Korth Kristalle GmbH, Altenholz (Kiel), Germany] was incubated with 300 μM NHS‐silane (Schartner *et al*, 2013), functionalised with 100 ng/ml monoclonal antibody A8978 (Sigma‐Aldrich (Munich, Germany) and saturated with an 1% w/v casein (Sigma‐Aldrich) blocking solution. All non‐specifically bound compounds were rinsed out with PBS buffer. The solutions were passed over the IRE surface by a peristaltic pump (Ismatec Reglo Digital, IDEX Health&Science, Wertheim‐Mondfeld, Germany) with a steady and defined flow of 1 ml/min. Afterwards, the functionalised Germanium IRE was covered with 50 μl plasma in a circulating flow (1 ml/min) and rinsed with PBS buffer. The total volume of the flow‐through sensor unit including all connection tubes was determined as 400 μl. For each plasma sample, a freshly prepared sensor surface was used. Difference absorbance spectra of each preparative step were recorded and served as a qualitative internal control for the success of each step. The total time for all preparative steps and Aβ analysis amounted to four hours for six independent samples.

In order to determine the reproducibility of the assay, we investigated freeze–thaw properties of the samples for this study and repeated measurements of 150‐blinded ESTHER samples. The results showed a high reproducibility of the amide I band of blood heparin plasma samples (± 1 cm^−1^) for each participant (Spearman rank correlation coefficient *r*_s_ = 0.991).

Before determination of the amide I maximum position, all spectra were baseline corrected and adjusted based on spectral traces of atmospheric water vapour. A straight horizontal line was passed through the spectra at distinct wavenumbers (3,970 cm^−1^, 2,700 cm^−1^, 1,750 cm^−1^, 1,485 cm^−1^, 1,000 cm^−1^) to provide a linear baseline to be subtracted from the measured signal. Atmospheric water vapour was removed by scaled subtraction of a reference spectrum. Additionally, high‐frequency noise was adjusted by a Fourier low‐pass filter.

The sensor was functionalised and saturated as described above for the extraction of the total Aβ fraction out of the plasma sample. The recorded Aβ difference spectrum represents the secondary structure distribution of all Aβ isoforms present in the respective fluid. Pure α‐helical Aβ absorbs at 1,650 cm^−1^ and pure β‐sheet at 1,624 cm^−1^ (fig 1 of Nabers *et al* 2016a). The more β‐sheet Aβ isoforms that are immobilised on the probe surface, the larger the spectral downshift to lower frequencies of the maximum position of the amide I band (fig S‐5 and S‐6 of Nabers *et al* 2016a). Mixed forms show different absorbance maxima, indicative of the secondary structure distribution as outlined in detail in fig S‐5 of Nabers *et al* (2016a). Thus, the position of the amide I maximum is indicative of the content of β‐sheet‐enriched Aβ species in the total Aβ fraction. We automatically determined the position of the Aβ amide I band for each patient sample by an in‐house procedure programmed with Matlab 2015A (Mathworks). This results in an unbiased, label‐free, robust, simple method. AD patients show an increased content of soluble and β‐sheet‐enriched Aβ isoforms within the total soluble Aβ fraction from blood and, in an even more pronounced manner, from cerebrospinal fluid (fig 2 of Nabers *et al*, 2016a).

#### Apoe (ESTHER)

*APOE* epsilon alleles were determined based on allelic combinations of the SNPs rs7412 and rs429358 using predesigned TaqMan SNP genotyping assays (Applied Biosystems, Foster City, CA). Genotypes were analysed in an endpoint allelic discrimination read using the Bio‐RAD CFX Connect System (Bio‐Rad Laboratories, Hercules, CA) in the laboratory of the University of Halle, Department of Psychiatry, Psychotherapy and Psychosomatics.

#### CSF analysis (BioFINDER)

CSF Aβ42, Aβ40 and ttau were analysed using Euroimmun kits (EUROIMMUN AG, Lübeck, Germany) and ptau using INNOTEST kit (Fujirebio, Gent, Belgium). All measurements were performed by board‐certified laboratory technicians who were blinded to clinical data.

### Amyloid ([^18^F]‐flutemetamol) PET (BioFINDER)

All 80 study participants from the BioFINDER study underwent [^18^F]‐flutemetamol PET to visualise cerebral Aβ deposition ([^18^F]‐flutemetamol is approved by the Food and Drug Administration, and the European Medical Agency). PET/CT scanning of the brain was conducted with Philips Gemini PET scanners as previously described (Palmqvist *et al*, 2014). In brief, sum images taken 90–110 min post‐injection were analysed using NeuroMarQ software (GE Healthcare). A volume of interest (VOI) template was applied for the following nine bilateral regions: prefrontal, parietal, lateral temporal, medial temporal, sensorimotor, occipital, anterior cingulate, posterior cingulate/precuneus and a global neocortical composite region (Lundqvist *et al*, 2013). The standardised uptake value ratio (SUVR) was defined as the uptake in a VOI normalised for the cerebellar cortex uptake. We have previously established [^18^F]‐flutemetamol SUVR cut‐off > 1.42 for abnormally increased Aβ deposition (Dubois *et al*, 2014). All cognitively healthy study participants included in the present study had normal [^18^F]‐flutemetamol PET scans, and all patients studied that had MCI had abnormal [^18^F]‐flutemetamol PET scans, that is the latter group fulfilled the IWG‐2 criteria for prodromal AD (Dubois *et al*, 2014).

### Data availability

Data protection standards, which were part of the informed consent procedure of the ESTHER study, preclude the publication of the source data in publicly available repositories. Individual data access may be granted on request within a framework of scientific cooperation.

### Statistical analysis

#### ESTHER study

**Figure EV2:**
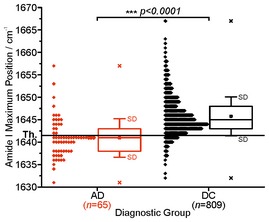
AD and disease control differentiation based on the Aβ secondary structure distribution in blood plasma (ESTHER) The Aβ amide I band absorbance maxima as recorded from heparin plasma samples from the years 2000–2002 (ESTHER) are shown as diamonds. The threshold (Th) at 1,642 cm^−1^ (solid horizontal line) separates AD (in red) and DC controls (in black). The biomarker discriminates with an accuracy of 88%. The DC control group consists of VD and MD cases, AD controls, VD controls and MD controls (see Fig 2). In box plots, 25/50/75% quantiles are shown as horizontal lines, the observed minimum/maximum values as (×), the average amide I band position as square and ± standard deviation as whiskers. Significant group differences are indicated by *P*‐values (two‐sided nonparametric Kruskal–Wallis analysis of variance test) and by asterisks: *P < 0.05, **P < 0.01, ***P < 0.001.

The distribution of the amide I biomarker band was compared between AD cases and AD controls. Additionally, the amide I band was also compared between the AD group and each of the other groups (VD and MD cases; AD, VD, and MD controls). Furthermore, we combined all of the non‐AD groups into a common disease control group (DC) (Fig EV2). Both diagnostic groups (AD, DC) did not show normally distributed amide I maxima. Thus, differences between groups were tested for statistical significance by a nonparametric Kruskal–Wallis analysis of variance test. Statistical tests were conducted two‐sided at a significance level of 0.05. Significance levels are denoted as follows: *P <* *0.05, **P <* *0.01, ***P <* *0.001.

Discrimination of AD from AD controls and DC was evaluated by receiver operator characteristics (ROC) curve analysis and quantified by the area under the curve. For ROC curve analyses, the threshold was set between 1,630.5 cm^−1^ and 1,659.5 cm^−1^, and for each wavenumber, the sensitivity and specificity were calculated. Sensitivity and specificity were determined assuming values of the amide I band below the threshold of 1,642 cm^−1^ to be indicative for AD. The classifying threshold of 1,642 cm^−1^ for AD and DC differentiation was determined based on a randomly selected patient collective as described in detail in Nabers *et al* (2016a). In that study, the performance of different thresholds was tested in repeated cross‐validations and verified with the remaining test‐data sets. Based on 30 repetitions, the study revealed 1,642 cm^−1^ as an optimal threshold for discrimination.

#### BioFINDER study

The detection, analysis of the amide I distribution and ROC curve analysis were performed in an identical manner to the ESTHER cohort analysis as presented above. In addition, the distribution of the amide I biomarker band was compared between the PET amyloid‐positive prodromal AD (MCI) and the PET amyloid‐negative control group.

#### Software

Statistical tests, tests for outliers, data preprocessing, receiver operating characteristic curve analysis and data distribution analysis were performed using Matlab 2012A, Matlab 2015A (Mathworks) and Origin 2015 (Origin Laboratories). In this context, built‐in and in‐house procedures programmed with Matlab were used, respectively.

## Author contributions

AN, LP, HB, OH and KG conceived the study. LP, UM and HB developed the NCC within the ESTHER study. OH developed the study design of the BioFINDER study. LP carried out the epidemiological analyses. AN and JL performed the immuno‐infrared analyses. JG and AN developed the sensor automatisation. JS synthesised reagents for the sensor functionalisation. AN carried out the immuno‐infrared data analyses. LP, AN, OH, SJ, HB and KG wrote the manuscript. UM, AN, HB, OH, SJ and KG interpreted the data. K‐US contributed to epidemiological analyses and to the coordination of the ESTHER study. BH and DR contributed to data acquisition for the ESTHER study. OH initiated PET scanning for the BioFINDER study. KG conceived the immuno‐infrared‐sensor for secondary structure analysis of protein misfolding. HB conceived and led the ESTHER study. All authors discussed the results, commented on the draft manuscript and approved the final manuscript.

## Conflict of interest

Dr. Hansson has received research support (for the institution) from Roche, GE Healthcare, Biogen, AVID Radiopharmaceuticals, Fujirebio and Euroimmun. In the past 2 years, he has received consultancy/speaker fees (paid to the institution) from Lilly, Roche and Fujirebio.

**The paper explained**

**Problem**

There is general agreement that early diagnosis of Alzheimer's disease (AD) based on minimal‐invasive and cost‐effective blood plasma methods will pave the way for future successful immuno‐therapies. Aβ misfolding into β‐sheet species occurs up to 20 years before clinical manifestation of AD and causes amyloid plaque formation in the brain.

**Results**

We applied an immuno‐infrared‐sensor that directly and label‐free monitors the Aβ secondary structure distribution in blood plasma. The assay works cost effective, simple and robust with only smallest amounts of blood plasma. The diagnostic read‐out is simply the secondary structure‐sensitive amide I frequency. AD is indicated by a frequency shift below the experimentally determined threshold. We applied this assay on baseline blood plasma in two different and independent cohorts on prodromal AD (BioFINDER, Sweden) and a population‐based longitudinal study (ESTHER, Germany). In these two cohorts, the assay shows a sensitivity around 70% and a specificity around 90%. In the BioFINDER study, there was a significant correlation between the secondary structure distribution of Aβ in blood plasma and Aβ burden in the brain monitored by [^18^F]‐flutemetamol positron emissions tomography (PET). Additionally, the Aβ misfolding biomarker showed also significant correlations with standard CSF biomarkers (Aβ42, Aβ42/40 ratio, total‐tau, phosphor‐tau) as already seen in our former published study on late stage AD. In the ESTHER study individuals with AD were identified in a nested case control group in average 8 years before clinical symptoms appeared.

**Impact**

Unique features of this assay are (i) the label‐free detection of the misfolding of Aβ in blood plasma, (ii) the simple procedure, (iii) the low sample volume and (iv) the low cost. Measuring the initial misfolding process which occurs 15–20 years before clinical manifestation, the immuno‐infrared‐assay may have the potential to detect AD earlier than Aβ burden tests. Moreover, there are indications that the sensor might also differentiate between AD and other dementia forms. This has to be validated in detail in larger studies. However, the immuno‐infrared‐sensor can also be applied to other neurodegenerative misfolding diseases such as Parkinson's disease.

## Supporting information



Appendix

Expanded View Figures PDF

Review Process File

Source Data for Figure 3
