# Supplementary material for: Amyloid blood biomarker detects Alzheimer's disease
Source: EMBO Mol Med. 2018 Apr 6;10(5):e8763. doi: 10.15252/emmm.201708763 (PMC5938617; doi:10.15252/emmm.201708763)
Supplement: Supplementary file 1 — Appendix [file EMMM-10-e8763-s001.pdf]

Appendix

AMYLOID BLOOD BIOMARKER DETECTS ALZHEIMER'S DISEASE

Andreas Nabers*[1], Laura Perna*[2], Julia Lange[1], Ute Mons[2], Jonas Schartner[1], Jörn Güldenhaupt[1], Kai-Uwe Saum[2], Shorena Janelidze[3], Bernd Holleczek[4], Dan Rujescu[5], Oskar Hansson[3,6], Klaus Gerwert[#2], Hermann Brenner[2,7]

*shared first authorship
# corresponding author

1) Ruhr-University Bochum – Department of Biophysics – 44780 Bochum, Germany
2) German Cancer Research Center (DKFZ) – Division of Clinical Epidemiology and Aging Research, Im Neuenheimer Feld 581 – 69120 Heidelberg, Germany
3) Department of Clinical Sciences, Lund University – 20502 Lund, Sweden
4) Saarland Cancer Registry, Präsident Baltz Straße 5 – 66119 Saarbrücken, Germany
5) University of Halle – Department of Psychiatry, Psychotherapy and Psychosomatics, Julius-Kühn-Str. 7 – 06112 Halle, Germany
6) Memory Clinic, Skåne University Hospital – 20502 Malmö, Sweden
7) Network Aging Research (NAR), University of Heidelberg, Bergheimer Straße 20 – 69115 Heidelberg, Germany

Table of Contents

- **Appendix Supplementary Figures**
  - Appendix Fig S1: In-silico calculation of the amide I band with different synthetic Aβ structural isoforms____________________________ **S.2**
  - Appendix Fig S2: Limit of quantification of the immuno-IR-assay __ **S.3**
  - Appendix Fig S3: Workflow of the immuno-IR-analysis__________ **S.4**
- **Appendix Supplementary Tables**
  - Appendix Tab S1: Baseline characteristics of ESTHER participants ____ **S.5**
  - Appendix Tab S2: Neurochemical and demographic data of the BioFINDER participants___________________________________ **S.6-7**
  - Appendix Tab S3: Mean follow-up time between blood collection and AD diagnosis (ESTHER)__________________________________ **S.8-9**

Appendix Supplementary Figure

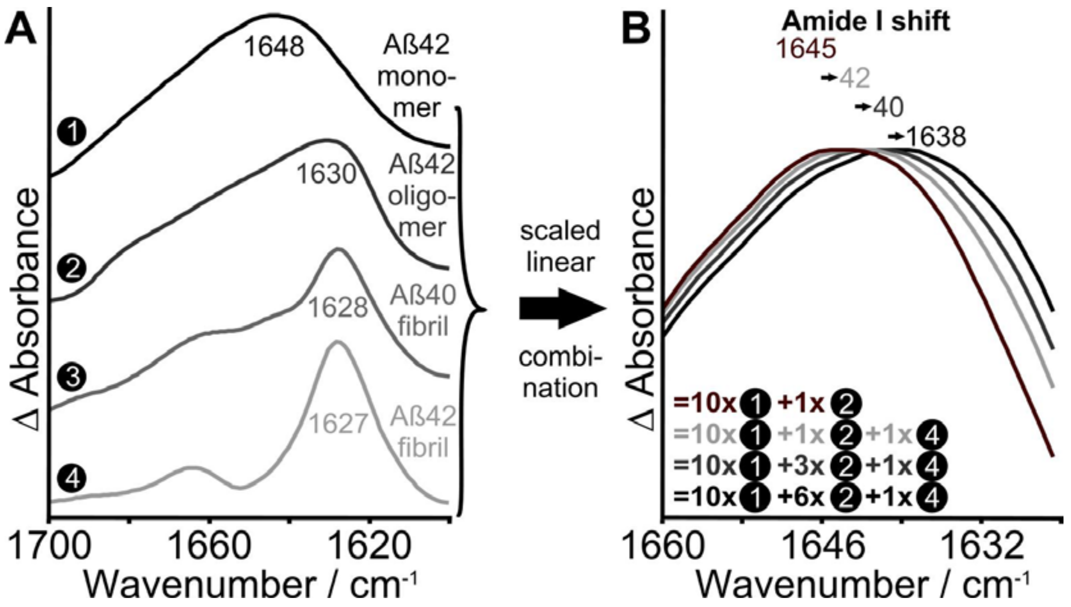

Appendix Figure S1: A: The antibody A8978 enables the extraction of synthetic Aß(42) monomers (1), Aß(42) oligomers (2), Aß(40) fibrils (3), and Aß(42) fibrils (4) from liquid samples. The amide I maximum depends on the respective secondary structure.

B: Scaled linear combinations of the synthetic Aß spectra (**a,** 1-4) with increasing ß-sheet content shift the amide I maximum towards lower wavenumbers, representative for the Aß fraction of AD cases.

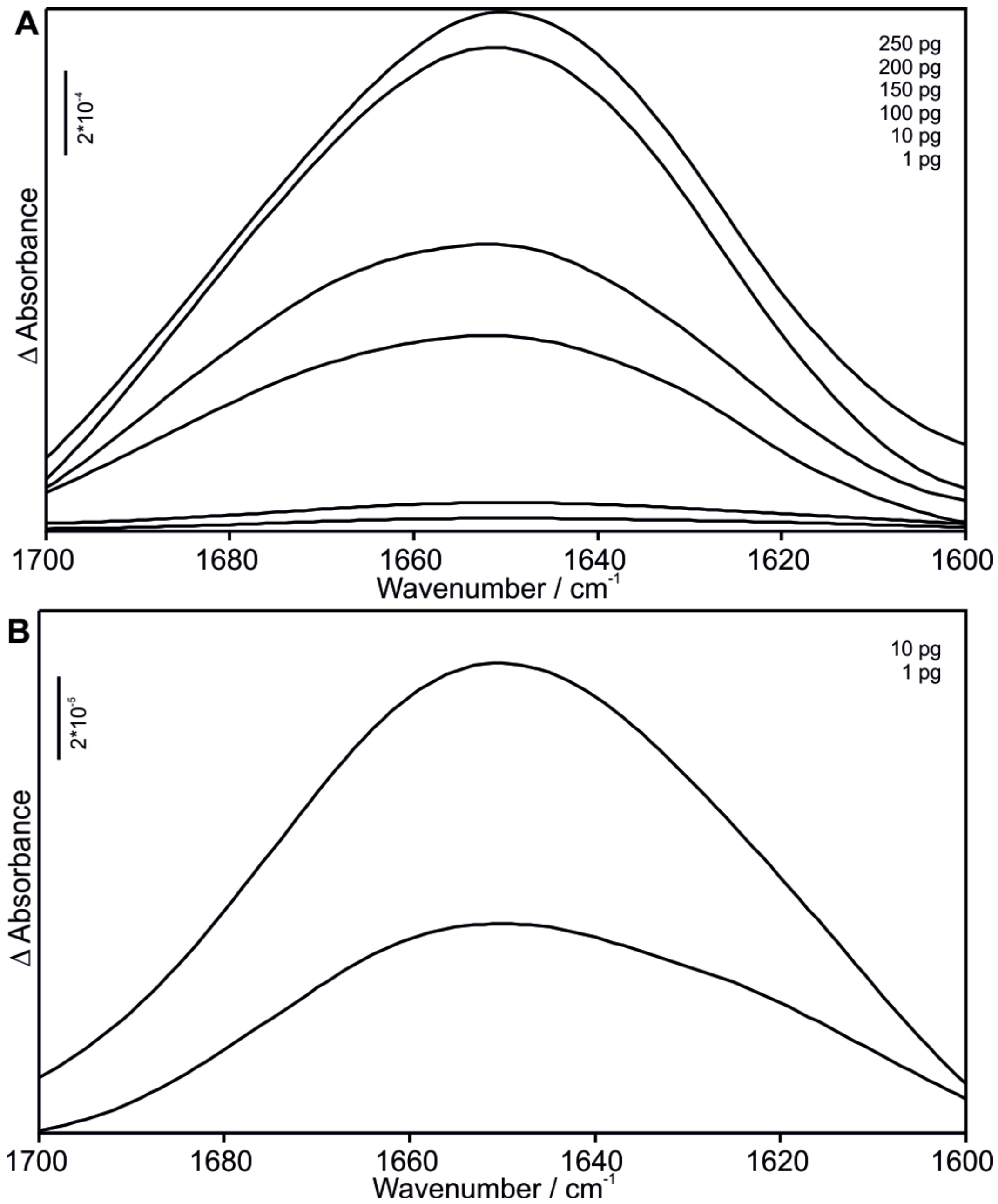

Appendix Figure S2: Limit of quantification of the immuno-IR-sensor for synthetic Aß(42).
A: The amide I band of antibody captured Aß was measured after adding synthetic Aß in different amounts (1 pg to 250 pg) to the total volume of the immuno-IR-system.
B: Even Aß amounts around 1 pg can be detected with a sufficient signal. All spectra were corrected from water vapour contributions and low-frequency noise.

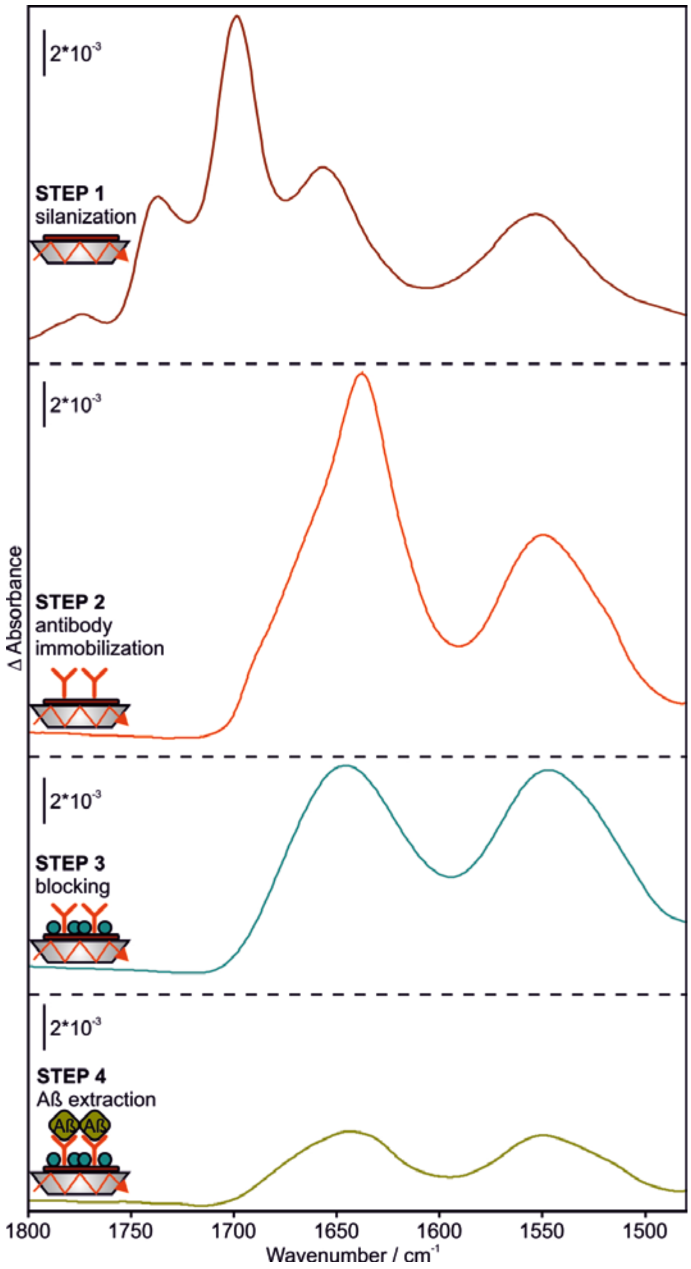

Appendix Figure S3: Workflow of the immuno-IR-analysis. In a first step, the internal reflection element is chemically functionalised by NHS-silanes. In this step, recorded difference absorbance spectra were calculated against the ATR-element and 2-Propanol as background. Unbound molecules were rinsed out with 2-Propanol accompanied by changing the medium from 2-Propanol to water and finally PBS buffer. After recording a new background (background: NHS-silane + buffer), the monoclonal antibody A8978 can be covalently bound to the surface, followed by rinsing with PBS buffer. In a third step, free reactive sites on the surface are saturated with a casein blocking solution (Background: NHS-silane + buffer + antibody A8978) and unbound molecules were rinsed out with buffer. Finally, Aß can be extracted from blood plasma in a fourth step (Background: NHS-silane + buffer + antibody + casein). Thereby, for each preparative step infrared difference spectra are recorded.

Appendix Table S1: Baseline characteristics of cases and controls* (ESTHER cohort study, 2000-2002).

	AD cases	AD controls	VD cases	VD controls	MD cases	MD controls
	n (%)	n (%)	n (%)	n (%)	n (%)	n (%)
**Sex**						
Women	40 (61.5)	154 (62.4)	42 (63.6)	185 (59.5)	15 (41.7)	68 (45.6)
Men	25 (38.5)	93 (37.6)	24 (36.4)	126 (40.5)	21 (58.3)	81 (54.4)
**Age****						
<65 years	11 (16.9)	45 (18.2)	13 (19.7)	81 (26.1)	2 (5.6)	12 (8.1)
≥65 years	54 (83.1)	202 (81.8)	53 (80.3)	230 (73.9)	34 (94.4)	137 (91.9)
**Education**						
≥10 years	9 (13.8)	40 (16.2)	3 (4.8)	17 (5.5)	6 (17.7)	27 (18.6)
≤9 years	56 (86.2)	207 (83.8)	59 (95.2)	294 (94.5)	28 (82.3)	118 (81.4)
**Amyloid test**						
≥ 1642	19 (29.2)	225 (91.1)	59 (89.4)	274 (88.1)	32 (88.9)	134 (89.9)
< 1642	46 (70.8)	22 (8.9)	7 (10.6)	37 (11.9)	4 (11.1)	15 (10.1)
ApoE* e4**						
Non-carriers[a)]	32 (54.2)	173 (78.3)	39 (68.4)	200 (73.8)	27 (79.4)	95 (73.6)
Carriers[b)]	27 (45.8)	48 (21.7)	18 (31.6)	71 (26.2)	7 (20.6)	34 (26.4)
**History of cardiovascular disease**						
No	57 (91.9)	204 (87.6)	48 (75.0)	259 (87.8)	25 (78.1)	128 (87.7)
Yes	5 (8.1)	29 (12.4)	16 (25.0)	36 (12.2)	7 (21.9)	18 (12.3)
**Diabetes mellitus**						
No	48 (73.9)	201 (81.4)	50 (76.9)	248 (80.5)	26 (74.3)	118 (79.7)
Yes	17 (26.1)	46 (18.6)	15 (23.1)	60 (19.5)	9 (25.7)	30 (20.3)
**History of cancer**						
No	59 (90.8)	222 (89.9)	62 (93.9)	280 (90.0)	33 (91.7)	128 (85.9)
Yes	6 (9.2)	25 (10.1)	4 (6.1)	31 (10.0)	3 (8.3)	21 (14.1)
**Smoking**						
Never/former	56 (87.5)	203 (85.7)	55 (87.3)	268 (89.6)	30 (88.2)	133 (91.1)
Current	8 (12.5)	34 (14.3)	8 (12.7)	31 (10.4)	4 (11.8)	13 (8.9)
**Physical activity**						
Medium/high[c)]	14 (21.5)	43 (17.4)	13 (19.7)	64 (20.6)	5 (13.9)	34 (23.0)
Low[d)]	27 (41.5)	131 (53.0)	30 (45.5)	154 (49.5)	15 (41.7)	64 (43.2)
Inactive[e)]	24 (36.9)	73 (29.6)	23 (34.8)	93 (29.9)	16 (44.4)	50 (33.8)
**BMI**						
<25 kg/m^2	25 (38.5)	63 (25.5)	14 (21.2)	67 (21.7)	8 (22.2)	32 (21.6)
25-29.9 kg/m^2	31 (47.7)	117 (47.4)	35 (53.0)	158 (51.1)	17 (47.2)	72 (48.7)
≥ 30 kg/m^2	9 (13.8)	67 (27.1)	17 (25.8)	84 (27.2)	11 (30.6)	44 (29.7)

*Cases and controls sum up to 874 (vs. 970) because only participants with Aβ measurements are represented. Hence cases and controls only approximately match. Variables not summing up to 874 include missing values in the original ESTHER dataset.
**Matching per age included age groups of approximately ± 3 years.
*** For technical reasons APOE e4 genotyping was not possible for 103 participants.
a) Participants not carrying APOE e4 genotype (e2e2, e2e3, e3e3)
b) Participants carrying APOE e4 genotype (e2e4, e3e4, e4e4)
c) ≥ 2 hours of vigorous and ≥ 2 hours of light physical activity /week
d) Other
e) < 1 hour of physical activity/week

Appendix Table S2: Phenotyping data of the patient cohort, including the dementia biomarkers, [^{18}F]-PET imaging results (SUVR), and the amide I maximum position of the Aβ fraction in blood plasma of DC (**A**) and MCI patients (**B**).

**A**<br>\#	age<br>[years]	gender<br>[male/female]	Aß(40)<br>[pg/ml]	Aß(42)<br>[pg/ml]	Aß(42/40)<br>[pg/ml]	ttau<br>[pg/ml]	ptau<br>[pg/ml]	[18F]-PET<br>[SUVR]	Amide I<br>[cm^{-1}]
1	73	female	5762	804	0.140	295	50	2.18	1641
2	77	female	3888	642	0.165	268	41	1.77	1641
3	70	female	6943	944	0.136	472	82	1.92	1645
4	71	female	5349	914	0.171	282	54	1.79	1647
5	80	female	3080	573	0.186	218	53	1.71	1643
6	82	female	8250	1089	0.132	280	63	1.83	1642
7	82	female	2983	606	0.203	159	33	1.16	1649
8	77	female	6535	1030	0.158	375	56	1.83	1642
9	78	female	5885	753	0.128	240	44	1.13	1646
10	66	male	5368	589	0.110	245	45	1.02	1647
11	79	female	7041	644	0.091	346	70	2.37	1644
12	77	male	4774	830	0.174	196	56	1.88	1647
13	72	female	4089	447	0.109	288	41	1.11	1643
14	66	male	5028	758	0.151	206	47	1.1	1642
15	77	female	5354	619	0.116	288	43	1.38	1645
16	81	female	3890	574	0.148	240	43	1.25	1645
17	76	female	3691	704	0.191	322	57	1.13	1646
18	81	male	7175	810	0.113	467	83	1.11	1644
19	72	male	4178	674	0.161	275	49	1.32	1643
20	71	male	2525	352	0.139	147	25	2.06	1641
21	73	female	2710	415	0.153	287	35	1.28	1643
22	73	female	2140	237	0.111	223	35	1.31	1643
23	69	male	3393	484	0.143	193	39	1.29	1643
24	69	male	5646	788	0.140	267	38	1.31	1646
25	78	male	7155	1111	0.155	464	77	1.35	1642
26	69	female	5409	646	0.119	499	78	1.26	1642
27	67	male	3548	414	0.117	267	41	1.29	1644
28	69	female	3130	463	0.148	245	38	1.34	1642
29	81	female	4699	575	0.122	330	52	1.2	1640
30	67	female	2506	427	0.170	155	27	1.04	1640
31	76	female	4532	603	0.133	280	56	1.19	1645
32	73	female	5317	579	0.109	331	60	1.16	1646
33	68	male	5125	515	0.100	268	54	1.29	1642
34	78	female	4634	693	0.150	336	60	1.21	1646
35	70	female	3647	527	0.145	272	44	1.11	1648
36	71	male	3993	688	0.172	278	38	1.18	1642
37	73	male	5491	804	0.146	359	55	1.26	1642
mean	74		4726	657	0.142	288	50	1.41	1644
SD ±	5		1499	200	0.026	85	14	0.35	2
min value	66		2140	237	0.091	147	25	1.02	1640
max value	82		8250	1111	0.203	499	83	2.37	1649

Appendix Table S2: Phenotyping data of the patient cohort, including the dementia biomarkers, [^{18}F]-PET imaging results (SUVR), and the amide I maximum position of the Aβ fraction in blood plasma of DC (**A**) and MCI patients (**B**).

**B**<br>#	age<br>[years]	gender<br>[male/female]	Aß(40)<br>[pg/ml]	Aß(42)<br>[pg/ml]	Aß(42/40)<br>[pg/ml]	ttau<br>[pg/ml]	ptau<br>[pg/ml]	[18F]-PET<br>[SUVR]	Amide I<br>[cm^{-1}]
38	77	female	6540	487	0.074	560	82	1.76	1647
39	77	female	4201	333	0.079	517	72	2.11	1640
40	65	male	3020	217	0.072	490	71	2.26	1640
41	71	male	4549	307	0.067	516	72	2.45	1641
42	77	female	5767	337	0.058	816	121	1.24	1641
43	79	male	4355	409	0.094	417	54	1.19	1640
44	68	female	2963	225	0.076	462	86	1.19	1646
45	77	female	5736	348	0.061	382	55	2.24	1640
46	75	female	5341	436	0.082	510	84	2.31	1647
47	70	male	5867	418	0.071	469	71	1.92	1640
48	76	female	7637	546	0.071	760	113	1.29	1644
49	71	female	5754	526	0.091	370	63	1.41	1639
50	76	male	3550	221	0.062	532	72	1.66	1640
51	78	male	5598	279	0.050	501	81	1.17	1639
52	74	male	5782	379	0.066	532	74	0.99	1640
53	67	male	5310	399	0.075	1136	174	2.12	1640
54	76	male	5867	409	0.070	400	78	1.72	1648
55	74	male	4918	219	0.045	619	101	1.21	1640
56	67	female	4412	374	0.085	452	79	1.23	1640
57	63	female	3860	237	0.061	355	54	1.14	1640
58	75	male	4972	340	0.068	341	50	2.36	1639
59	67	male	5739	444	0.077	552	88	1.59	1640
60	77	male	6228	549	0.088	596	66	2.67	1640
61	72	male	4915	280	0.057	316	71	2.37	1644
62	72	male	5966	312	0.052	657	112	1.79	1645
63	71	male	3116	251	0.081	437	60	1.73	1642
64	68	male	6484	473	0.073	539	106	1.88	1639
65	79	male	2929	230	0.079	231	33	2.41	1642
66	62	female	5651	303	0.054	567	75	1.99	1641
67	80	male	8217	594	0.072	493	84	1.44	1640
68	80	female	7276	531	0.073	775	120	1.44	1640
69	78	male	3144	264	0.084	368	43	2.3	1639
70	66	female	3902	285	0.073	516	67	2.84	1639
71	73	male	3868	290	0.075	366	70	2.13	1647
72	75	female	7848	408	0.052	1324	209	2.07	1643
73	62	male	7700	401	0.052	903	167	2.66	1641
mean	73		5250	363	0.070	549	86	1.84	1641
SD ±	5		1463	107	0.012	221	37	0.51	3
min value	62		2929	217	0.045	231	33	0.99	1639
max value	80		8217	594	0.094	1324	209	2.84	1648

Appendix Table S3: The time period in years from recruitment to dementia diagnosis and the corresponding amide I maximum of AD, VD, and MD cases from the ESTHER cohort study. Time period data were available from 58 AD (of 65), 60 VD (of 66), and 35 MD (of 36) cases.

AD			VD			MD		
#	infrared-amide I Maximum [cm^{-1}]	years from recruitment to dementia diagnosis	#	infrared-amide I Maximum [cm^{-1}]	years from recruitment to dementia diagnosis	#	infrared-amide I Maximum [cm^{-1}]	years from recruitment to dementia diagnosis
1	1646	6	59	1658	9	119	1654	11
2	1638	12	60	1641	5	120	1645	11
3	1645	10	61	1645	11	121	1646	4
4	1635	9	62	1643	4	122	1645	10
5	1636	9	63	1642	4	123	1650	12
6	1638	12	64	1642	9	124	1646	12
7	1638	10	65	1642	11	125	1635	9
8	1640	4	66	1648	10	126	1644	8
9	1645	10	67	1642	10	127	1644	9
10	1636	10	68	1645	7	128	1645	10
11	1636	13	69	1645	2	129	1647	6
12	1657	4	70	1642	10	130	1644	11
13	1640	8	71	1652	14	131	1646	11
14	1641	9	72	1641	9	132	1662	11
15	1641	12	73	1656	12	133	1642	11
16	1641	8	74	1648	9	134	1644	10
17	1636	2	75	1647	6	135	1645	10
18	1650	9	76	1652	7	136	1643	11
19	1638	14	77	1647	9	137	1639	10
20	1641	8	78	1643	9	138	1658	4
21	1640	5	79	1640	5	139	1647	9
22	1644	2	80	1645	8	140	1644	14
23	1646	11	81	1642	10	141	1645	5
24	1640	4	82	1644	12	142	1640	7
25	1640	8	83	1643	10	143	1645	5
26	1642	7	84	1646	9	144	1648	8
27	1638	10	85	1658	12	145	1644	4
28	1640	8	86	1649	11	146	1651	6
29	1642	9	87	1646	4	147	1646	11
30	1648	4	88	1643	7	148	1641	1
31	1636	6	89	1644	0	149	1647	6
32	1640	7	90	1645	12	150	1644	3
33	1637	6	91	1646	9	151	1644	12
34	1641	6	92	1644	11	152	1656	6
35	1638	9	93	1645	8	153	1643	7
36	1643	12	94	1644	13			
37	1641	7	95	1649	6			
38	1641	10	96	1647	9			
39	1641	8	97	1645	12			
40	1644	5	98	1643	12			
41	1641	5	99	1643	11			
42	1634	6	100	1645	4			
43	1638	9	101	1641	13			
44	1641	8	102	1642	12			
45	1644	14	103	1642	13			
46	1645	8	104	1643	13			
47	1641	11	105	1640	11			
48	1638	10	106	1645	10			
49	1637	13	107	1644	13			
50	1641	6	108	1642	12			
51	1631	6	109	1645	12			
52	1643	9	110	1644	14			
53	1653	4	111	1650	9			
54	1641	5	112	1653	12			
55	1641	12	113	1642	4			
56	1640	3	114	1650	13			
57	1637	8	115	1644	5			
58	1641	9	116	1649	6			
			117	1642	13			
			118	1642	6			
mean	1641	8		1645	9		1646	8
SD±	4	3		4	3		5	3
