# Supplementary material for: Amyloid blood biomarker detects Alzheimer's disease
Source: EMBO Mol Med. 2018 Apr 6;10(5):e8763. doi: 10.15252/emmm.201708763 (PMC5938617; doi:10.15252/emmm.201708763)
Supplement: Supplementary file 2 — Expanded View Figures PDF [file EMMM-10-e8763-s002.pdf]

Expanded View Figures

	vs.	r_s	*p* value
amide I maximum	Aß(42)	0.401	4×10^{-4}
	Aß(40)	-0.039	0.7443
	Aß(42/40)	0.407	4×10^{-4}
	total Tau	-0.382	8×10^{-4}
	phospho-Tau	-0.172	0.1463
	age	0.112	0.3442
	SUVR (^{18}F PET)	-0.397	5×10^{-4}

Figure EV1. Correlation between Aβ from blood plasma and CSF or PET (BioFINDER).

Summary of the Spearman rank correlation coefficients (r_s) and their *P*-values between the amide I maximum positions of Aβ from EDTA plasma, neurochemical biomarkers from CSF and SUVR values from ^{18}F PET imaging.

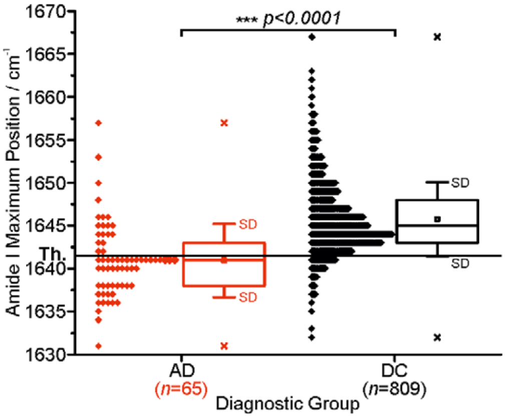

Figure EV2. AD and disease control differentiation based on the Aβ secondary structure distribution in blood plasma (ESTHER).

The Aβ amide I band absorbance maxima as recorded from heparin plasma samples from the years 2000–2002 (ESTHER) are shown as diamonds. The threshold (Th) at 1,642 cm^{-1} (solid horizontal line) separates AD (in red) and DC controls (in black). The biomarker discriminates with an accuracy of 88%. The DC control group consists of VD and MD cases, AD controls, VD controls and MD controls (see Fig 2). In box plots, 25/50/75% quantiles are shown as horizontal lines, the observed minimum/maximum values as (×), the average amide I band position as square and ± standard deviation as whiskers. Significant group differences are indicated by *P*-values (two-sided nonparametric Kruskal–Wallis analysis of variance test) and by asterisks: *$P < 0.05$, **$P < 0.01$, ***$P < 0.001$.

© 2018 The Authors
