# Supplementary material for: Amyloid blood biomarker detects Alzheimer's disease
Source: EMBO Mol Med. 2018 Apr 6;10(5):e8763. doi: 10.15252/emmm.201708763 (PMC5938617; doi:10.15252/emmm.201708763)
Supplement: Supplementary file 3 — Review Process File [file EMMM-10-e8763-s004.pdf]

EMBO Molecular Medicine

Amyloid blood biomarker detects Alzheimer's disease

Andreas Nabers, Laura Perna, Julia Lange, Ute Mons, Jonas Schartner, Jörn Güldenhaupt, Kai-Uwe Saum, Shorena Janelidze, Bernd Holleczek, Dan Rujescu, Oskar Hansson, Klaus Gerwert, Hermann Brenner

Review timeline:

Submission date:	12 December 2017
Editorial Decision:	9 February 2018
Revision received:	20 February 2018
Editorial Decision:	26 February 2018
Revision received:	2 March 2018
Accepted:	6 March 2018

Editor: Céline Carret

Transaction Report:

(Note: Please note that the manuscript was transferred from another journal where it was originally reviewed. Since the original reviews are not subject to EMBO's transparent review process policy, the reports and author response cannot be published.)

© European Molecular Biology Organization

EMBO PRESS

YOU MUST COMPLETE ALL CELLS WITH A PINK BACKGROUND

PLEASE NOTE THAT THIS CHECKLIST WILL BE PUBLISHED ALONGSIDE YOUR PAPER

Corresponding Author Name: Prof. Dr. Klaus Gerwert

Journal Submitted to: EMBO Molecular Medicine
Manuscript Number: EMM-2017-08763

Reporting Checklist For Life Sciences Articles (Rev. June 2017)

This checklist is used to ensure good reporting standards and to improve the reproducibility of published results. These guidelines are consistent with the Principles and Guidelines for Reporting Preclinical Research issued by the NIH in 2014. Please follow the journal's authorship guidelines in preparing your manuscript.

A- Figures
1. Data
The data shown in figures should satisfy the following conditions:

- the data were obtained and processed according to the field's best practice and are presented to reflect the results of the experiments in an accurate and unbiased manner.
- figure panels include only data points, measurements or observations that can be compared to each other in a scientifically meaningful way.
- graphs include clearly labeled error bars for independent experiments and sample sizes. Unless justified, error bars should not be shown for technical replicates.
- if n< 5, the individual data points from each experiment should be plotted and any statistical test employed should be justified
- Source Data should be included to report the data underlying graphs. Please follow the guidelines set out in the author ship guidelines on Data Presentation.

2. Captions

Each figure caption should contain the following information, for each panel where they are relevant:

- a specification of the experimental system investigated (eg cell line, species name).
- the assay(s) and method(s) used to carry out the reported observations and measurements
- an explicit mention of the biological and chemical entity(ies) that are being measured.
- an explicit mention of the biological and chemical entity(ies) that are altered/varied/perturbed in a controlled manner.

- the exact sample size (n) for each experimental group/condition, given as a number, not a range;
- a description of the sample collection allowing the reader to understand whether the samples represent technical or biological replicates (including how many animals, litters, cultures, etc.).
- a statement of how many times the experiment shown was independently replicated in the laboratory.
- definitions of statistical methods and measures:
  - common tests, such as t-test (please specify whether paired vs. unpaired), simple χ2 tests, Wilcoxon and Mann-Whitney tests, can be unambiguously identified by name only, but more complex techniques should be described in the methods section;
  - are tests one-sided or two-sided?
  - are there adjustments for multiple comparisons?
  - exact statistical test results, e.g., P values = x but not P values < x;
  - definition of 'center values' as median or average;
  - definition of error bars as s.d. or s.e.m.

Any descriptions too long for the figure legend should be included in the methods section and/or with the source data.

In the pink boxes below, please ensure that the answers to the following questions are reported in the manuscript itself. Every question should be answered. If the question is not relevant to your research, please write NA (non applicable).
We encourage you to include a specific subsection in the methods section for statistics, reagents, animal models and human subjects.

USEFUL LINKS FOR COMPLETING THIS FORM

http://www.antibodypedia.com
http://1degreebio.org
http://www.equator-network.org/reporting-guidelines/improving-bioscience-research-repo

http://grants.nih.gov/grants/olaw/olaw.htm
http://www.mrc.ac.uk/Ourresearch/Ethicsresearchguidance/Useofanimals/index.htm
http://ClinicalTrials.gov
http://www.consort-statement.org
http://www.consort-statement.org/checklists/view/32-consort/66-title
http://www.equator-network.org/reporting-guidelines/reporting-recommendations-for-tum
http://datadryad.org
http://figshare.com
http://www.ncbi.nlm.nih.gov/gap
http://www.ebi.ac.uk/ega
http://biomodels.net/
http://biomodels.net/miriam/
http://jjj.biochem.sun.ac.za
http://oba.od.nih.gov/biosecurity/biosecurity_documents.html
http://www.selectagents.gov/

B- Statistics and general methods

Please fill out these boxes (Do not worry if you cannot see all your text once you press return)

1.a. How was the sample size chosen to ensure adequate power to detect a pre-specified effect size?	Within the ESTHER cohort we designed a nested-case control (NCC) study and included all reported AD, VD, and MD cases as of January 2016, where the response rate of the GPs reached a plateau. We randomly selected the controls from strata defined by age, sex, educational level as matching factors (frequency matching), so that cases and controls were similar with regard to these relevant confounding factors. In order to increase statistical power we chose more than one control per case, but we did not exceed the 4:1 ratio, since it has been demonstrated that a further increase of the ratio only leads to trivial improvements in precision and power29. Controls were selected randomly from the whole ESTHER cohort using the "surveyselect" procedure included in the statistical software SAS® version 9.4 (SAS® Institute Inc., Cary, NC, USA) (page 13).<br>In the BioFINDER cohort forty cognitively healthy elderly, who were amyloid ([18F]-flutemetamol) PET negative, and 40 non-demented patients with mild cognitive impairment, who were amyloid PET positive, were included in the present study (page 14).<br>The sample size was calculated by a sample size calculator for a given specificity of 70% in a pilot study including 10 MCI vs. 10 Controls. This calculation resulted in group sizes of 40 vs. 40.
1.b. For animal studies, include a statement about sample size estimate even if no statistical methods were used.	NA
2. Describe inclusion/exclusion criteria if samples or animals were excluded from the analysis. Were the criteria pre-established?	The exclusion of patient samples for the immuno-infrared-analysis due to insufficient performance in the assay or insufficient sample quality is described in the manuscript. Further inclusion and exclusion criteria for study participants are explained in the online methods.
3. Were any steps taken to minimize the effects of subjective bias when allocating animals/samples to treatment (e.g. randomization procedure)? If yes, please describe.	All samples in this study were blinded (see point 5) so no randomization was performed.
For animal studies, include a statement about randomization even if no randomization was used.	NA
4.a. Were any steps taken to minimize the effects of subjective bias during group allocation or/and when assessing results (e.g. blinding of the investigator)? If yes please describe.	The assay was performed in a blinded manner (page 5,8,14,18). The investigators did the infrared-analysis at the Biophysics department at the Ruhr-University Bochum. After data interpretation in Bochum and sending the results to the DKFZ Heidelberg the study was deblinded.
4.b. For animal studies, include a statement about blinding even if no blinding was done	NA
5. For every figure, are statistical tests justified as appropriate?	Confirmed

Do the data meet the assumptions of the tests (e.g., normal distribution)? Describe any methods used to assess it.	The data did not follow a normal distribution. Therefore, we used Spearman rank correlations and nonparametric Kruskal-Wallis tests for the quantification and analysis of significance. We avoid the calculation of postive predictive values because in the BioFINDER cohort both diagnostic groups were of the same size. We calculated likelihhod ratios as additional parameter for sensitivity, and specificity.
Is there an estimate of variation within each group of data?	The experimantal findings were fist obtained on the ESTHER samples and aftwards reproduced on the BioFINDER samples. Both datasets are completely independent from each other. In order to determine the reproducibility of the assay we investigated freeze-thaw properties of the samples for this study and repeated measurements of 150-blinded ESTHER samples. The results showed a high reproducibility of the amide I band of blood heparin-plasma samples (+/- 1 cm-1) for each participant (Spearman rank correlation coefficient rs=0.991).
Is the variance similar between the groups that are being statistically compared?	yes, see also Figure 2, extended data, and result description within the manuscript text.

C- Reagents

6. To show that antibodies were profiled for use in the system under study (assay and species), provide a citation, catalog number and/or clone number, supplementary information or reference to an antibody validation profile. e.g., Antibodypedia (see link list at top right), 1DegreeBio (see link list at top right).	We used monoclonal antibody A8978 from sigma-aldrich (Germany). This antibody is a commercial product. We used the same antibody in a former publication on Alzheimer's disease detection.
7. Identify the source of cell lines and report if they were recently authenticated (e.g., by STR profiling) and tested for mycoplasma contamination.	NA

* for all hyperlinks, please see the table at the top right of the document

D- Animal Models

8. Report species, strain, gender, age of animals and genetic modification status where applicable. Please detail housing and husbandry conditions and the source of animals.	NA
9. For experiments involving live vertebrates, include a statement of compliance with ethical regulations and identify the committee(s) approving the experiments.	NA
10. We recommend consulting the ARRIVE guidelines (see link list at top right) (PLoS Biol. 8(6), e1000412, 2010) to ensure that other relevant aspects of animal studies are adequately reported. See author guidelines, under 'Reporting Guidelines'. See also: NIH (see link list at top right) and MRC (see link list at top right) recommendations. Please confirm compliance.	NA

E- Human Subjects

11. Identify the committee(s) approving the study protocol.	BioFINDER: Regional Ethics Committee in Lund, Sweden; ESTHER: approved by the Ethics Committee of the Medical Faculty of the University of Heidelberg and of the Physicians' Board of Saarland
12. Include a statement confirming that informed consent was obtained from all subjects and that the experiments conformed to the principles set out in the WMA Declaration of Helsinki and the Department of Health and Human Services Belmont Report.	All participants gave written informed consent in both studies (BioFINDER and ESTHER).
13. For publication of patient photos, include a statement confirming that consent to publish was obtained.	NA
14. Report any restrictions on the availability (and/or on the use) of human data or samples.	NA
15. Report the clinical trial registration number (at ClinicalTrials.gov or equivalent), where applicable.	NA
16. For phase II and III randomized controlled trials, please refer to the CONSORT flow diagram (see link list at top right) and submit the CONSORT checklist (see link list at top right) with your submission. See author guidelines, under 'Reporting Guidelines'. Please confirm you have submitted this list.	NA
17. For tumor marker prognostic studies, we recommend that you follow the REMARK reporting guidelines (see link list at top right). See author guidelines, under 'Reporting Guidelines'. Please confirm you have followed these guidelines.	NA

F- Data Accessibility

18: Provide a "Data Availability" section at the end of the Materials & Methods, listing the accession codes for data generated in this study and deposited in a public database (e.g. RNA-Seq data: Gene Expression Omnibus GSE39462, Proteomics data: PRIDE PXD000208 etc.) Please refer to our author guidelines for 'Data Deposition'.<br><br>Data deposition in a public repository is mandatory for:<br>a. Protein, DNA and RNA sequences<br>b. Macromolecular structures<br>c. Crystallographic data for small molecules<br>d. Functional genomics data<br>e. Proteomics and molecular interactions	Data protection standards, which were part of the informed consent procedure of the ESTHER study, preclude the publication of the source data in publicly available repositories. Individual data access may be granted on request within a framework of scientific cooperation. The authors declare no competing financial interests.
19. Deposition is strongly recommended for any datasets that are central and integral to the study; please consider the journal's data policy. If no structured public repository exists for a given data type, we encourage the provision of datasets in the manuscript as a Supplementary Document (see author guidelines under 'Expanded View' or in unstructured repositories such as Dryad (see link list at top right) or Figshare (see link list at top right).	Data are available in the Extended data.
20. Access to human clinical and genomic datasets should be provided with as few restrictions as possible while respecting ethical obligations to the patients and relevant medical and legal issues. If practically possible and compatible with the individual consent agreement used in the study, such data should be deposited in one of the major public access-controlled repositories such as dbGAP (see link list at top right) or EGA (see link list at top right).	Data are available in the Extended data.
21. Computational models that are central and integral to a study should be shared without restrictions and provided in a machine-readable form. The relevant accession numbers or links should be provided. When possible, standardized format (SBML, CellML) should be used instead of scripts (e.g. MATLAB). Authors are strongly encouraged to follow the MIRIAM guidelines (see link list at top right) and deposit their model in a public database such as Biomodels (see link list at top right) or JWS Online (see link list at top right). If computer source code is provided with the paper, it should be deposited in a public repository or included in supplementary information.	NA

G- Dual use research of concern

22. Could your study fall under dual use research restrictions? Please check biosecurity documents (see link list at top right) and list of select agents and toxins (APHIS/CDC) (see link list at top right). According to our biosecurity guidelines, provide a statement only if it could.	NA
